# Prolonged Maternal Separation Reduces Anxiety State and Increases Compulsive Burying Activity in the Offspring of BALB/c Mice

**DOI:** 10.3390/jpm12111921

**Published:** 2022-11-17

**Authors:** Qais Jarrar, Rami Ayoub, Kawther Alhussine, Khang Wen Goh, Said Moshawih, Chrismawan Ardianto, Bey Hing Goh, Long Chiau Ming

**Affiliations:** 1 Department of Applied Pharmaceutical Sciences and Clinical Pharmacy, Faculty of Pharmacy, Isra University, Amman 11622, Jordan; 2 Faculty of Data Science and Information Technology, INTI International University, Nilai 71800, Malaysia; 3 PAPRSB Institute of Health Sciences, Universiti Brunei Darussalam, Gadong BE1410, Brunei Darussalam; 4 Department of Pharmacy Practice, Faculty of Pharmacy, Universitas Airlangga, Surabaya 60115, Indonesia; 5 Biofunctional Molecule Exploratory Research Group, School of Pharmacy, Monash University Malaysia, Petaling Jaya 47500, Malaysia; 6 College of Pharmaceutical Sciences, Zhejiang University, Hangzhou 310058, China

**Keywords:** maternal separation, anxiety, compulsivity, adaptation, reproductive health, pregnancy, marble burying test, mental disease, depression, impulsivity

## Abstract

Background: The elevated plus maze (EPM) and the marble burying (MB) tests are common behavioral tests used for behavioral phenotyping in mouse models for neurodevelopmental disorders. However, the behavioral effects of maternal separation (MS), a standard paradigm for early life stress in animals, in both the EPM and MB tests remain incompletely known. Objectives: This study aimed to investigate the behavioral effects of prolonged MS in the offspring of mice using the EPM and MB tests. Methods: Male BALB/c mice were isolated from their mothers for 4 h each day during the first 30 days after birth. On day 50 postnatal, groups of separated and non-separated mice (*n* = 18/each group) were subjected to the EPM and MB tests for comparative behavioral evaluations. In addition, the locomotor activity of mice was evaluated using the actophotometer test. Results: The findings of the EPM test revealed that separated mice exhibited anxiolytic-like behaviors, as evidenced by a significant increase in the latency to closed arms and the time spent in the open arms compared with non-separated mice. Separated mice also showed compulsive burying activity in the MB test, as determined by a significant increase in the number of buried marbles. The results of the actophotometer test did not show any significant change in locomotor activity. Conclusions: Prolonged MS caused the adult offspring of mice to exhibit a decrease in anxiety state and increased compulsive burying activity, which were not associated with a change in locomotor activity. Further investigations with validated tests are needed to support these findings.

## 1. Introduction

Early childhood adversity (CA) refers to a condition in which individuals are exposed to a variety of stressors early in life, particularly during childhood [1]. Adverse childhood experiences include several types of traumatic events, such as physical, emotional and sexual abuse, as well as physical and emotional neglect, lack of maternal care and poverty [2,3]. Although the prevalence rate of childhood trauma differs substantially based on adversity type, gender, culture and continent, it can affect more than 10% of the general population [4]. According to research based on questionnaires from community samples around the world, 75% of respondents had experienced childhood trauma, with a mean of three incidents [5]. In addition, statistics have found that patients with anxiety, obsessive-compulsive disorder (OCD) and other mental disorders present more frequently with a history of childhood trauma than healthy populations [6].

There is considerable evidence in the research suggesting that CA has a complex and non-specific effect on body systems that are associated with multiple health complications on an individual’s physiology, behaviors and neural functions [7]. These effects are predisposing factors to chronic diseases such as hypertension, diabetes mellitus and cancer, or can be associated with mental disorders [8], deviant behavior [9] and psychopathology [7]. Previous studies have also suggested that the adverse effects of CA present at any time during the lifespan, even later in adulthood, with long-lasting and permanent effects [10]. Nevertheless, the severity of CA complications is believed to be influenced by various rearing conditions, such as the time, intensity and duration of exposure to the adversity.

Neuroimaging and microscopy studies have found that CA can alter brain morphology and causes a wide variety of structural, volumetric and demyelinating effects in various neural regions in the brain [11,12,13,14]. Cumulative studies have also shown that CA can impair the functional connectivity between various neural regions, including the connection between the amygdala and prefrontal cortex, which is involved in regulating the anxiety state [15,16]. There is a line of evidence that shows that CA results in a diminished anxiety state in adult offspring [17,18]. Some researchers suggested that a reduced anxiety state may be attributed to a decrease in the innate anxiety response that is related to the connection of the amygdala and prefrontal cortex [19,20]. However, other research groups have suggested that the anxiolytic effect is due to an impulsive action that triggers individuals to engage in risky behaviors [21,22].

Although genetic factors play a major role in the development of OCD [23], environmental factors substantially contribute to its etiology [24]. There is a sizable amount of clinical research showing a causal link between CA and the development of OCD. These studies indicate that patients with OCD are more frequently associated with a history of CA than healthy individuals [25]. Additionally, the severity of OCD symptoms is positively correlated with the number of early CA [26,27]. Notably, the effects on corticocortical and limbic circuits caused by CA are similar to those seen in OCD, suggesting a pathophysiological relationship [28].

One potential negative childhood experience is the separation from one’s mother. It has been suggested that early separations impair the mother’s ability to form an attachment, which reduces the possibility that the child and mother will develop a secure relationship [29]. The correlation between maternal separation (MS) and aberrant behaviors in humans was recognized early on by John Bowlby, who suggested that continual disruption of the attachment between infants and mothers was associated with a high risk of cognitive impairment and affectionless psychopathy [30,31]. Later studies found that children living in institutions with little opportunity for caregiver–child interaction grew more slowly in terms of their physical, cognitive and social–emotional abilities and had a higher rate of behavioral and executive function issues, even years after adoption [32,33]. Epidemiological studies also showed that early MS is associated with a higher risk of developing schizotypal personality disorder symptoms [34], frequent nightmares [35] and alcohol and drug dependence in early adulthood [36]. Moreover, there is evidence that MS causes differential impacts on fear-related behaviors in a sex- and age-specific way [37].

During the last few decades, animal research has been extensively used to investigate CA’s effects, with a greater emphasis on the MS paradigm [38]. From an experimental viewpoint, MS is defined as a procedure employed to isolate newborns from their mothers for a predetermined period, which might range from a few hours to many days. A brief and short separation procedure entails a daily separation of 15 min over a period that can be extended to 14 days [39]. In contrast, a severe and prolonged separation procedure entails a daily separation of 3–4 h, which lasts for more than 14 days [40]. Several studies have shown that the MS consequence varies based on multiple variables, such as the time and duration of separation, as well as the animal species and strains used in different research groups [41,42,43,44,45]. However, there is a significant body of evidence from studies in animals that indicates that MS can disrupt hypothalamus–pituitary–adrenal axis function [46], induce long-term oxidative stress in the brain [47] and cause lasting changes in emotion-related behaviors, as well as impairing the growth of areas that are involved in the stressful stimuli [48]. Neurochemical disorders [49], spatial memory loss [50], impulsivity [51] and risk-taking behaviors such as drug abuse [52] were also correlated to the effects of MS.

The EPM and MB tests are common behavioral tests used for examining anxiety-like behaviors in animals [53,54]. The MB test is also indicated for examining compulsive-like behaviors [55,56] Although the EPM has frequently been used to investigate the behavioral effects of MS, results obtained from this test are inconsistent among different research groups. While many studies found that MS can induce anxiety-like behaviors in the EPM test [57,58], a few others found that MS resulted in reduced anxiety-like behaviors [59]. This suggests that the experimental conditions of the MS paradigm are not well standardized in animal studies, which necessitates additional studies for conclusive evidence. In addition, there is a glaring paucity of empirical research showing the role of MS in participating in OCD. In light of this, the present study aimed to extend the previous studies by investigating the behavioral effect of severe and prolonged MS in multimodal tests, including the EPM, MB and locomotor activity tests. We hypothesized, in the current study, that the use of such a multimodal approach could aid in a better understanding of the behavioral effects of MS.

## 2. Methods

### 2.1. Animals Care and Husbandry

BALB/c mice were used as the model of choice in this study. All mice were kept under well-controlled conditions (room temperature = 22–25 °C and relative humidity = 50–52% in standard 12 h light/dark cycles). Mice had unrestricted access to tap water and standardized food pellets. Mice were raised in a noise-free environment at Isra University’s animal facility in Jordan. All animal handling and use procedures were approved by the Institution of Scientific Research Ethics of Isra University (1/4-2021/2022).

### 2.2. Maternal Separation Protocol

The procedure of MS was conducted as described previously [60,61,62] with some modifications. Female pregnant mice were distributed individually into clean, transparent cages 3–6 days before giving birth. After birth, mice mothers were separated from their pups for 4 h daily (between 9 a.m. and 1 p.m.) for 30 days or were left undisturbed (in the case of the control group), only handled during cage cleaning. During separation, mice mothers were placed in separate cages with clean bedding and free access to food pellets and drinking water. After day 30 postnatal, pups were kept in their cages and raised with their dams and siblings, without any separation. All mice (dams and pups) were kept at room temperature (22–25 °C) and relative humidity (50–52%). Control mice (non-separated offspring) were subjected to the same conditions, without exposure to the MS procedure. At day 50 postnatal, adult male mice were submitted to a variety of behavioral tests, including the EPM, MB and the actophotometer tests. The behavioral tests were repeated in three different sets of animals (*n* = 6/each group/each set). Data obtained from maternally separated mice were compared with their counterparts of non-separated mice. All behavioral tests were conducted at the Laboratory of Behavioral Pharmacology at Isra University, Jordan.

### 2.3. Elevated plus Maze Test

The EPM apparatus was used as described previously [63]. The apparatus was composed of a “+”-shaped maze elevated above the floor (60 cm in height) with four arms (two oppositely positioned closed arms and two oppositely positioned open arms of 55 cm length and 10 cm width) emerged from a center square. The test began with each mouse being placed in the center area while its head was facing the open arm. The latency to closed arms, the amount of time spent in the closed and open arms, the total arm entries and the number of head-dips were recorded over a 5 min period. All anxiety-like behaviors were recorded using a digital camera and scored by an expert observer who was blind to the experimental groups.

### 2.4. Marble Burying Test

Plastic transparent cages filled with 5-cm-thick burying material of wood chips (density 0.13 g/cm^3^ and average diameter 0.6 cm) and provided with glass marbles (20 mm diameter) were employed, as described previously [55]. The marbles (*n* = 20) were uniformly spaced on the surface of the burying material of each cage and arranged in lines (parallel to each other). The experiment began with each mouse being placed in its cage, while the burying behavior was tracked by counting the total number of marbles buried at 15 and 30 min during the experiment. The marble was considered buried when at least two thirds of its original volume was covered with wood chips.

### 2.5. Measurement of Locomotor Activity

The actophotometer model was used to quantify spontaneous coordinate activity in mice, as previously described [64]. The test began with the placement of each mouse individually in the animal activity cage of clear Perspex (Ugo Basile, 40 × 40 cm) for the measurement of horizontal movement over 10 min throughout the experiment. The number of infrared beam interruptions was automatically counted by the actophotometer and was measured as an index of locomotor activity.

### 2.6. Statistical Analysis

Data obtained from animal tests were presented as mean ± standard error of the mean (SEM). Statistical significance between groups was calculated by the unpaired t test using the GraphPad Prism software. A *p*-value less than 0.05 was considered significant between animal groups.

## 3. Results

### 3.1. Elevated plus Maze Test

**Figure 1:**
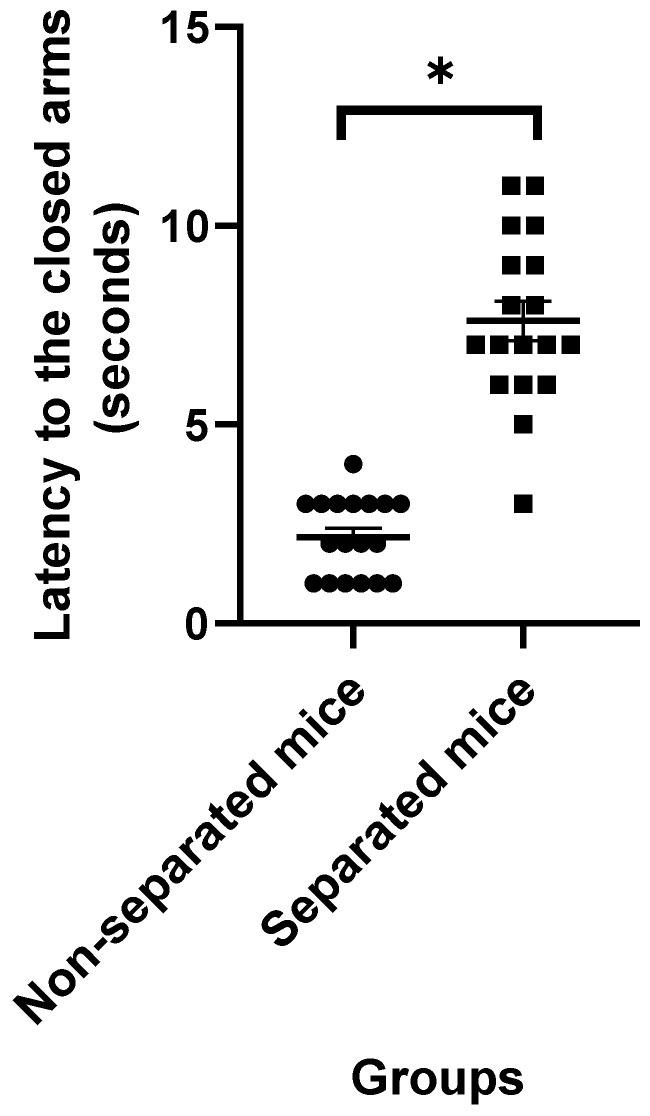
Latency to the closed arms. (*) indicates significant difference (*p* < 0.05) between separated and non-separated mice by the unpaired *t* test (*n* = 18/each group).

**Figure 2:**
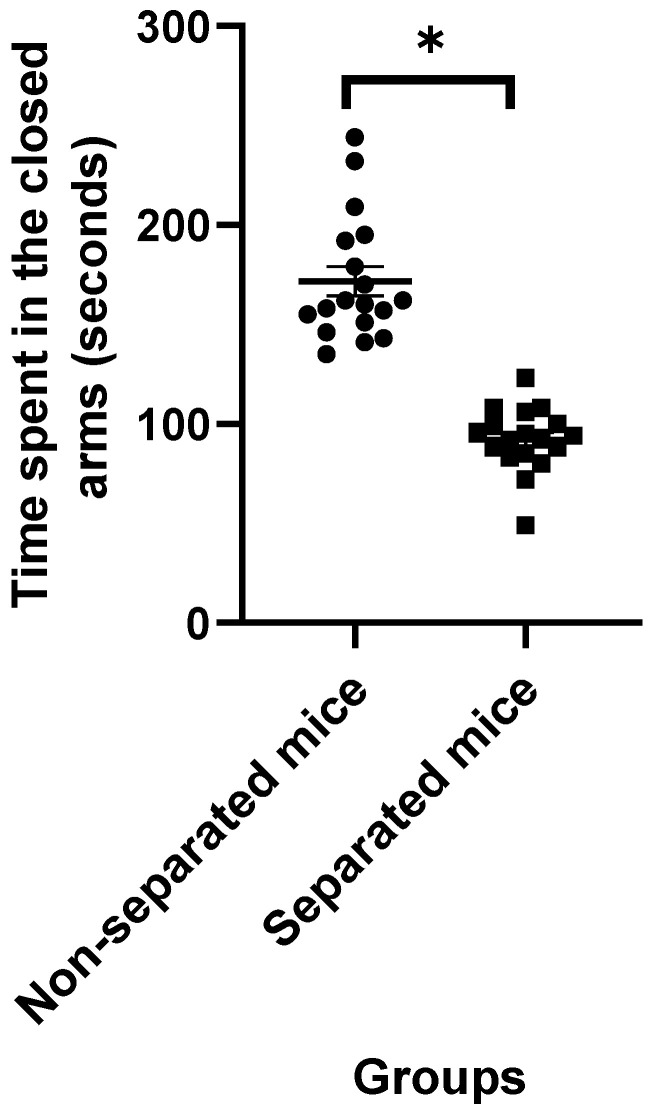
Time spent in the closed arms. (*) indicates significant difference (*p* < 0.05) between separated and non-separated mice by the unpaired *t* test (*n* = 18/each group).

**Figure 3:**
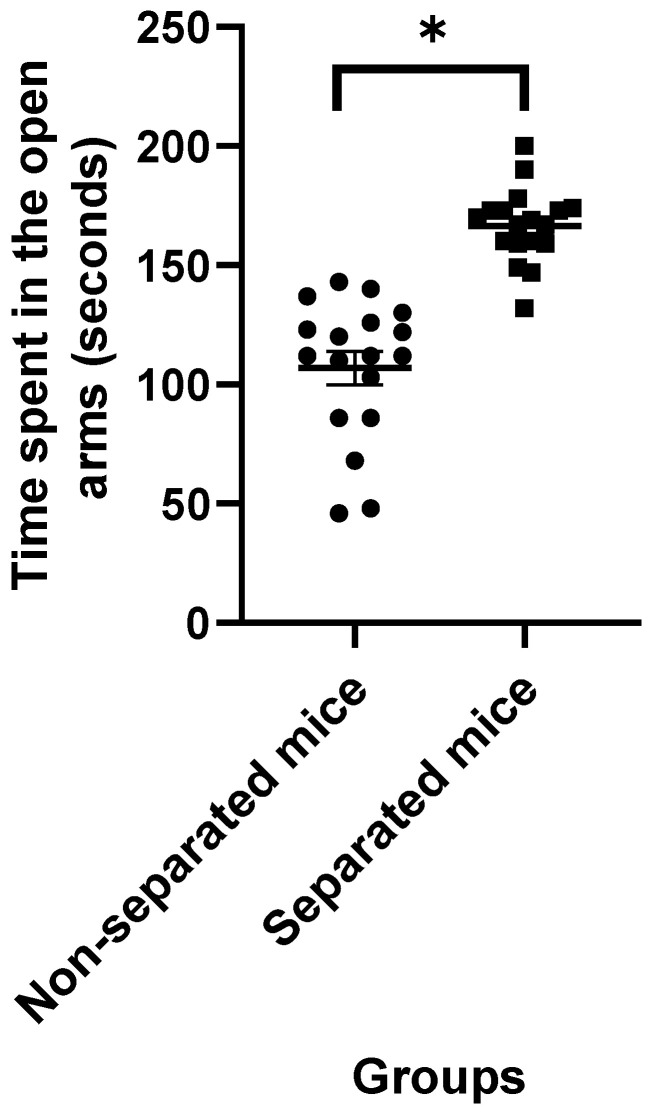
Time spent in the open arms. (*) indicates significant difference (*p* < 0.05) between separated and non-separated mice by the unpaired *t* test (*n* = 18/each group).

**Figure 4:**
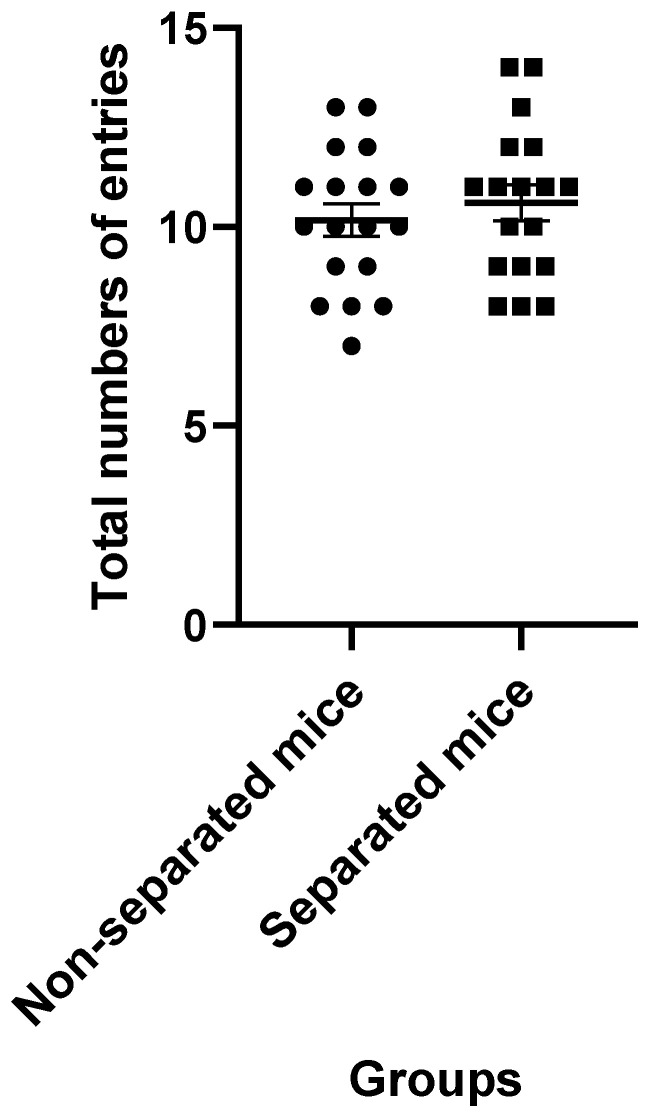
Total arm entries in the EPM test. There was no significant difference between the groups (*p* > 0.05) (*n* = 18/each group).

**Figure 5:**
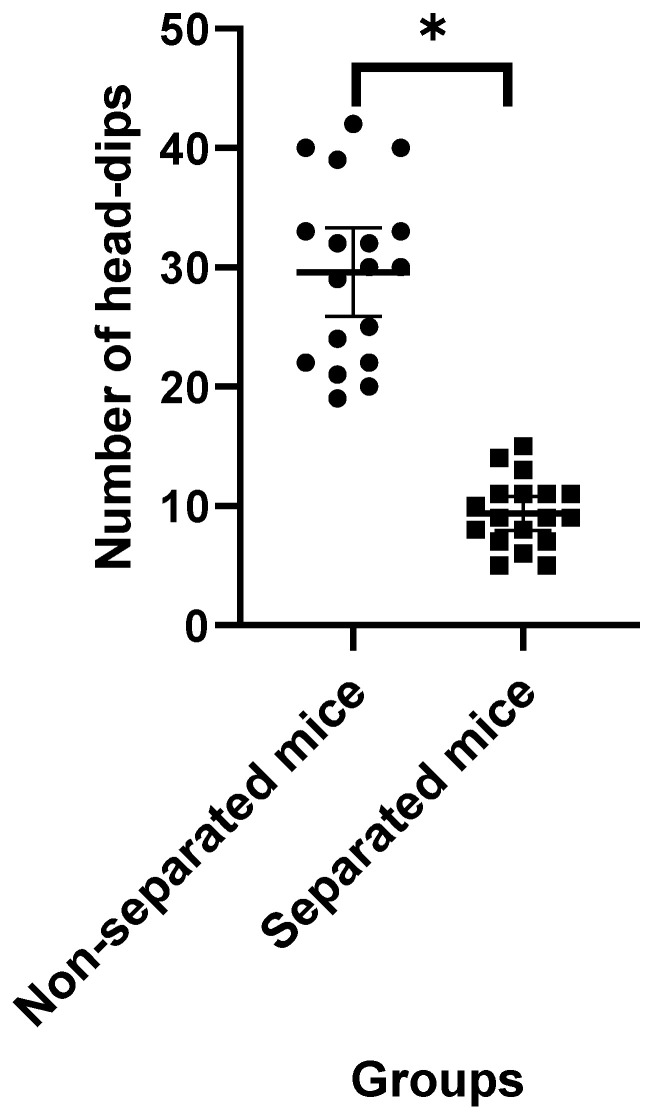
Number of head-dips in experimental groups. (*) indicates significant difference (*p* < 0.05) between separated and non-separated mice by the unpaired *t* test (*n* = 18/each group).

Data obtained from the EPM test revealed that mice challenged with repeated MS exhibited higher latency to the closed arms (Figure 1) and a significant increase in the amount of time spent in the closed and open arms (Figure 2 and Figure 3, respectively) compared to their non-separated counterparts. No significant change (*p* = 0.47) was observed in the total number of entries to the arms between the separated and non-separated mice (Figure 4). The results also showed that the total number of head-dips was significantly decreased in the separated mice (Figure 5). A total of 36 mice were used in this test (*n* = 18/group).

### 3.2. Marble Burying Test

**Figure 6:**
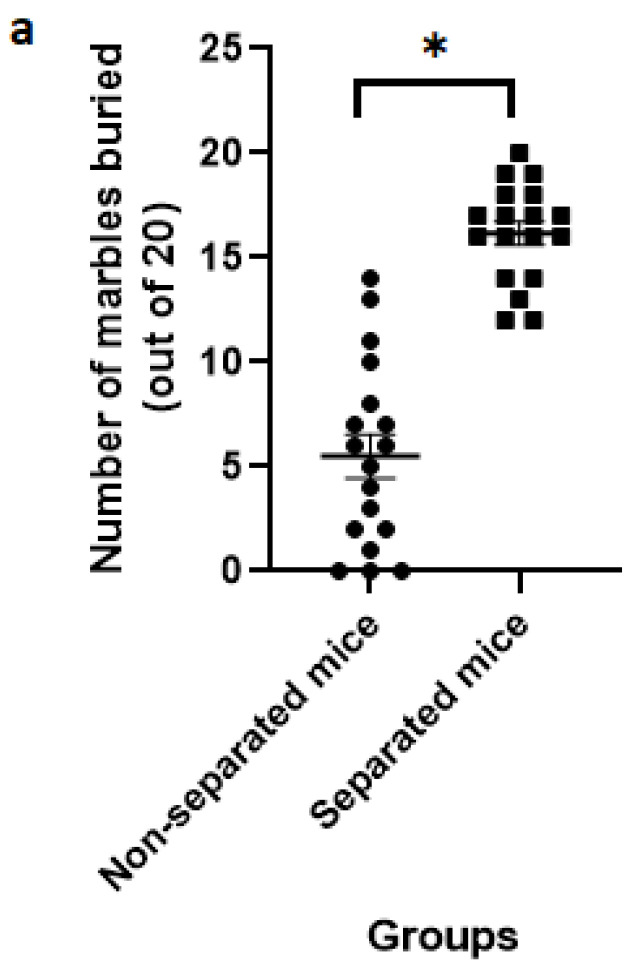

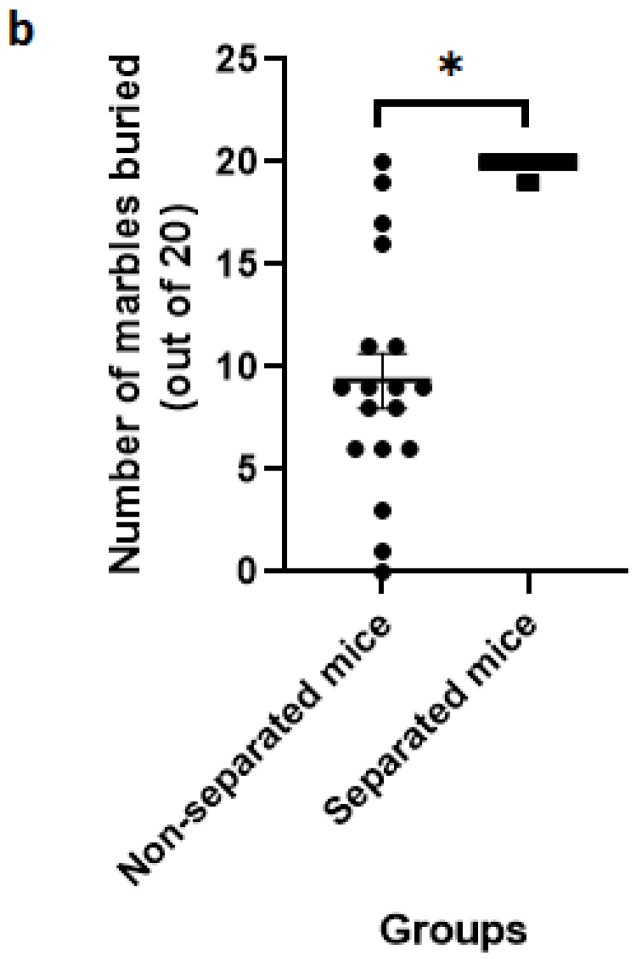
(**a**) Number of marbles buried at 15 min during MB test. (**b**) Number of marbles buried at 30 min during MB test. (*) indicates significant difference (*p* < 0.05) between separated and non-separated mice by the unpaired *t* test (*n* = 18/each group).

The findings from the repeated MB test revealed that separated mice buried a significantly higher number of marbles than non-separated mice at 15 and 30 min of the experimental session (Figure 6). A total of 36 mice were used in this test (*n* = 18/group). Interestingly, the intra-individual variation among the separated mice was less than that observed in the non-separated counterparts.

### 3.3. Measurement of Locomotor Activity

**Figure 7:**
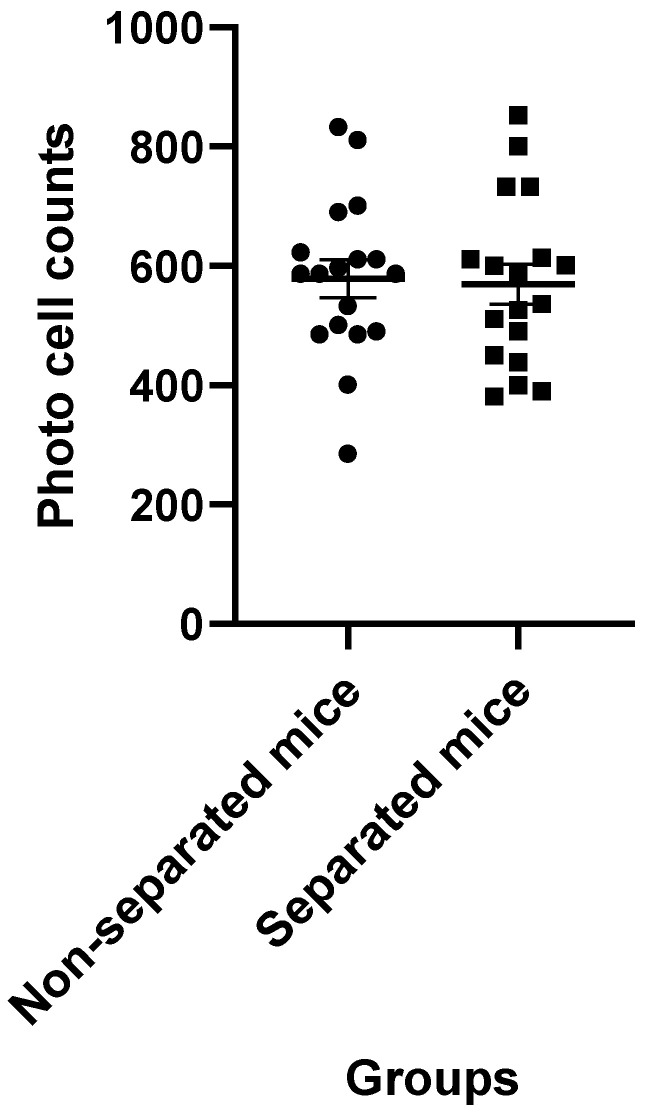
Average of locomotor activity of maternally separated mice as measured by the number of light beam interruptions compared to the non-separated mice (*n* = 18/each group). There was no significant difference between the groups (*p* > 0.05).

Data obtained from the actophotometer showed that separated mice had comparable horizontal movement to that of non-separated mice. The statistical analysis failed to find any significant difference (*p* = 0.8) between these groups (Figure 7). A total of 36 mice were used in this test (*n* = 18/group).

## 4. Discussion

Over the past few decades, there has been mounting evidence that adverse childhood experiences can affect how individuals respond to both disease and treatment [65]. Many studies showed that childhood adversity can cause epigenetic changes [66] and increase an individual’s susceptibility to various illnesses, such as cancer, heart disease, endocrine disorders and mental illnesses [67,68]. Likewise, the findings of the current study provide supporting evidence that mice exposed to early life stress by being separated from their mothers (from day 1 to 30 postnatal) were more likely to exhibit aberrant behaviors than their non-separated peers. These behaviors were characterized by a significant decrease in anxiety state and a significant increase in compulsive-like behaviors. Therefore, MS can influence the inter-individual variation in the susceptibility to diseases, such as anxiety and compulsive-related disorders, and their pharmacotherapy.

Because different MS paradigms were used in animal studies, including the use of different species, strains, time and duration of separation, the findings of behavioral alterations were inconsistent. Some studies showed that MS resulted in cognitive impairments [69], anxiety [70] and depressive like-behaviors [44] in rodents. However, other studies reported coping, risk-taking and paradoxical behavioral effects [71]. These results indicate that mother–child interactions are highly susceptible to various environmental factors, which can alter brain development in particular ways.

Although most previous studies focused on the effects of maternal separation during infancy and the early weaning period (from day 1 to 21 postnatal), this study, however, was designed to examine the effect of a prolonged separation procedure that covered the prepubertal period (from day 1 to 30 postnatal). Research on brain development suggested that the maturing brain, during the prepubertal and adolescence period, may be particularly vulnerable to the effect of stress, and this effect may significantly increase the likelihood of developing risky behaviors later in life [72] and cause differential impacts on fear-related behaviors in a sex- and age-specific way [37]. Similarly, this study showed evidence that prolonged MS reduced the anxiety level, which reflects risk-taking behavior. In addition, this is the first study that aimed to investigate the effect of prolonged maternal separation on the marble burying activity in the offspring of mice.

Coping with various forms of early childhood stress has been recognized as an important element of life that promotes healthy development [73]. However, the capacity to cope with stress can be affected by a number of factors, including the type and severity of the stress [74]. Studies indicate that strong and frequent stress might have a negative impact on a child’s growth and brain architecture [75]. Therefore, identifying distinct types of stress and understanding their effects may aid in making better decisions regarding the need for interventions that lower the risk of long-term unfavorable outcomes. In this study, a prolonged MS procedure was employed to identify the behavioral effect of maternal care deprivation on BALB/C mice. In addition, a combination of multiple behavioral tests, including the EPM, MB and actophotometer tests, was used to analyze the complex and multifaceted effects of prolonged MS in the BALB/c mice. All behavioral tests were repeated in three different sets of animals to verify the validity of the results from the separation procedure employed in this study.

BALB/c mice are a very common inbred strain in biological research that is frequently used in behavioral studies [76,77]. According to recent studies, these mice are generally more susceptible to social defeat stress than C57BL/6 mice, another popular mouse strain, and exhibit a greater anxious response and less social activity [41,78,79]. Moreover, these mice showed a phenotypic trait of compulsivity that can be exacerbated over exposure to chronic stress [80,81,82]. Although previous studies showed that different mice strains showed distinctive stress sensitivity and anxiety-like behaviors [83], the effect of prolonged MS on BALB/c mice remains unknown. These together may justify the use of the BALB/c strain in the current study. In addition, female mice were not used for the behavioral tests because male and female siblings were not separated at any time before the experiments. Therefore, using adult female mice (at age 50 days) was not appropriate due to possible concerns about the effect of sexual maturation and potential gestation. Sibling separation was avoided in this study as the primary aim was to examine the negative impact of maternal separation and to rule out the possible effects inflicted by other stress factors, such as the separation of siblings.

The use of the EPM test is a valid method to assess anxiety in laboratory mice [84,85]. The principle function of this test is essentially based on the tendency of mice to display normal aversion to open spaces while exhibiting a greater preference to return to the closed areas to avoid potential threats [86]. The measuring parameters of the EPM test may include anxiety-related measures (e.g., the time spent in the closed arms), impulsivity-related measures (e.g., the time spent in the open arms) and locomotion-related behaviors (e.g., the total entries to the arms). A previous study showed that anxiety measures in the EPM test were downregulated by benzodiazepines, whereas serotonin receptor agonists showed an anxiolytic effect in only one measure [87]. This may indicate that benzodiazepine receptors have a key role in regulating anxiety-like behaviors in the EPM test. In this study, data from the EPM test revealed that, contrary to our expectation, separated mice had less anxiety than their non-separated counterparts, as demonstrated by a significant increase in the latency to the closed arms and a significant decrease in the time spent in the closed arms. In addition, these mice exhibited decreased head-dipping behavior, which is a potential escape response in mice [88]. The reduction in the anxiety state in response to prolonged MS may indicate that BALB/c mice have a high capacity to cope with chronic stress. Previous studies have suggested that the reduction in the anxiety state in the MS paradigm is attributed to an adaptogenic mechanism that aids individuals in coping with stress [21]. They hypothesized that repeated exposure to early life stress can accelerate the maturation in the connection between the amygdala and prefrontal cortex, which in turn leads to a progressive decrease in cortisol reactivity to stress [20,89]. Another study has suggested that the adaptogenic effect following CA could be attributed to a change in the central dopaminergic activity [21].The separated mice also showed impulsive-like behavior in the EPM test, which was determined by a significant increase in the amount of time spent in the open arms. Impulsivity, or impulsive action, refers to the inability to inhibit one’s behavioral drives and thoughts, which results in activities that are poorly conceived, prematurely expressed, excessively risky or inappropriate for the situation and frequently have unintended consequences. Epidemiological studies have shown a positive association between CA and increased risk of impulsivity and engaging in risky behaviors [22,90].

The EPM data also revealed that total arm entries in the MS paradigm did not correlate with anxiety-related measures. This may indicate that, at least in part, prolonged MS reduced the anxiety state without causing a change in the locomotory activity of BALB/c mice. This was compatible with the result of the actophotometer test, which did not show any significant difference in the locomotor activity between separated and non-separated peers.

Burying behavior, which is defined as the process of placing the bedding materials over noxious and harmless objectives, is a normal behavior in wild and laboratory rodents [91,92]. The motivations underlying the burying behavior are multi-faceted, including the tendency to store food for hoarding, protect against noxious (e.g., glass marbles) and harmful objects and provide an appropriate environment for breeding. Several studies have suggested that MB behavior in mice, which is a common animal model in behavioral research, has a compulsive nature [55,56] that may resemble mild compulsive behaviors in humans. However, recent studies have suggested that excessive burying behavior is a valid prediction of compulsive-related disorders [93,94]. Compulsive disorder has been defined as an aberrant tendency to perform an action persistently and repetitively that occurs as an attempt to mitigate or distract from obsessive thoughts [95]. It has been proposed that mice treat glass marbles as harmful objects and they bury them repetitively to eliminate such a source of threat [56]. The findings of the present study revealed that separated mice showed a significant increase in the number of buried marbles at various times throughout repeated experimental sessions, indicating that these mice exhibited compulsive-like behaviors. The findings of the MB test provide empirical support for the clinical evidence describing an association between CA and the development of OCD [26,96]. Therefore, the prolonged MS paradigm can be used as a useful tool for elucidating the neural basis that underlies the pathogenesis of OCD.

The results of the present study lend at least some support to attachment theory [97], which suggests that the mother–child attachment system, particularly in the early years, functions as a key component in regulating children’s emotions and behaviors and mediating healthy development in later life. Although this study has reached its aims, there were some unavoidable limitations. First, this research was restricted to exploring the alterations in behavioral phenotypes. Therefore, further studies are needed that involve some biochemical and molecular evaluations. Specifically, as there is a line of evidence showing that cortisol reactivity is sensitive to a wide variety of environmental stressors and gradually changes over time during body development, measuring cortisol levels may therefore aid in understanding how the MS procedure used in the current study contributes in decreasing the anxiety state and increasing the compulsive-like behaviors in mice [46,98,99,100]. Additionally, further studies are needed to determine how the MS method used in this study affects the connectivity between the amygdala and prefrontal cortex, as a growing body of research suggests that mother–child attachment has significant effects on the regulation and maturation of this neurocircuitry, which is crucial for forming normal behaviors and healthy emotions. Second, our results could not determine whether the behavioral alterations in this study could be attributed to the effect of MS itself or whether they were indirectly related to the stress inflicted on the mothers, which could affect the care given to their pups. A line of emerging evidence demonstrated that poorer environmental quality was linked to altered breastfeeding behaviors and breast milk composition, as well as a reduced likelihood of breastfeeding initiation and a higher risk of terminating nursing [101,102]. This could be addressed in future studies by examining maternal care measures. Finally, as the male and female mice were not separated in the current study, the behavioral tests were conducted only on the male mice to avoid concerns related to the effect of sex maturation and possible gestation. Additional studies are required to elaborate the potential differences between the sexes to understand the influence of early MS in later developing psychopathology.

## 5. Conclusions

The results of this study offer empirical support for previous studies that found an association between MS and the emergence of aberrant behaviors in mice. Prolonged MS caused adult offspring of mice to exhibit a decrease in anxiety states and increased compulsive burying activity, which were not associated with a change in locomotor activity. Impulsivity appears to mediate reduced anxiety, although more behavioral and biological measurements, as well as assessing the difference in both males and females using rigorously validated tests, are needed to support these findings.

## References

[1] Nakamura J.S., Kim E.S., Rentscher K.E., Bower J.E., Kuhlman K.R. (2022). Early-life stress, depressive symptoms, and inflammation: The role of social factors. Aging Ment. Health.

[2] Offerman E.C.P., Asselman M.W., Bolling F., Helmond P., Stams G.-J.J.M., Lindauer R.J.L. (2022). Prevalence of adverse childhood experiences in students with emotional and behavioral disorders in special education schools from a multi-informant perspective. Int. J. Environ. Res. Public Health.

[3] Sharratt K., Mason S.J., Kirkman G., Willmott D., McDermott D., Timmins S., Wager N.M. (2022). Childhood abuse and neglect, exposure to domestic violence and sibling violence: Profiles and associations with sociodemographic variables and mental health indicators. J. Interpers. Violence.

[4] Moody G., Cannings-John R., Hood K., Kemp A., Robling M. (2018). Establishing the international prevalence of self-reported child maltreatment: A systematic review by maltreatment type and gender. BMC Public Health.

[5] Pace C.S., Muzi S., Rogier G., Meinero L.L., Marcenaro S. (2022). The Adverse Childhood Experiences–International Questionnaire (ACE-IQ) in community samples around the world: A systematic review (part I). Child Abuse Negl..

[6] Lochner C., du Toit P.L., Zungu-Dirwayi N., Marais A., van Kradenburg J., Seedat S., Niehaus D.J.H., Stein D.J. (2002). Childhood trauma in obsessive-compulsive disorder, trichotillomania, and controls. Depress. Anxiety.

[7] Oginga F.O., Magwai T., Shangase K.B., Xulu K.R., Mpofana T. (2022). Early Life Stress and Brain Plasticity: From Alterations of Brain Morphology to Development of Psychopathology. NeuroSci.

[8] Zovetti N., Perlini C., Brambilla P., Bellani M. (2022). Childhood adversities and bipolar disorder: A neuroimaging focus. Epidemiol. Psychiatr. Sci..

[9] Rachel C., Roman N.V., Donga G.T. (2022). The Contribution of Parental Factors to Adolescents’ Deviant Behaviour in South Africa: Evidence from Three Rural Communities in South Africa. Soc. Sci..

[10] Nilaweera D., Freak-Poli R., Gurvich C., Ritchie K., Chaudieu I., Ancelin M.-L., Ryan J. (2022). The association between adverse childhood events and later-life cognitive function and dementia risk. J. Affect. Disord..

[11] Marusak H.A., Kuruvadi N., Vila A.M., Shattuck D.W., Joshi S.H., Joshi A.A., Jella P.K., Thomason M.E. (2016). Interactive effects of BDNF Val66Met genotype and trauma on limbic brain anatomy in childhood. Eur. Child. Adolesc. Psychiatry.

[12] Calem M., Bromis K., McGuire P., Morgan C., Kempton M.J. (2017). Meta-analysis of associations between childhood adversity and hippocampus and amygdala volume in non-clinical and general population samples. Neuroimage Clin..

[13] Brooks S.J., Dalvie S., Cuzen N.L., Cardenas V., Fein G., Stein D.J. (2014). Childhood adversity is linked to differential brain volumes in adolescents with alcohol use disorder: A voxel-based morphometry study. Metab. Brain Dis..

[14] Gasecka A., Lutz P.-E., Tanti A., Mechawar N., Turecki G., Côté D.C. Early Life Adversity Leads to Demyelination in the Anterior Cingulate Cortex. Proceedings of the Novel Techniques in Microscopy; Optical Society of America.

[15] Park A.T., Leonard J.A., Saxler P.K., Cyr A.B., Gabrieli J.D.E., Mackey A.P. (2018). Amygdala–medial prefrontal cortex connectivity relates to stress and mental health in early childhood. Soc. Cogn. Affect. Neurosci..

[16] Herringa R.J., Burghy C.A., Stodola D.E., Fox M.E., Davidson R.J., Essex M.J. (2016). Enhanced prefrontal-amygdala connectivity following childhood adversity as a protective mechanism against internalizing in adolescence. Biol. Psychiatry Cogn. Neurosci. Neuroimaging.

[17] Fontana B.D., Cleal M., Norton W.H.J., Parker M.O. (2021). The impact of chronic unpredictable early-life stress (CUELS) on boldness and stress-reactivity: Differential effects of stress duration and context of testing. Physiol. Behav..

[18] Qin X., He Y., Wang N., Zou J.-X., Zhang Y.-M., Cao J.-L., Pan B.-X., Zhang W.-H. (2019). Moderate maternal separation mitigates the altered synaptic transmission and neuronal activation in amygdala by chronic stress in adult mice. Mol. Brain.

[19] Callaghan B.L., Sullivan R.M., Howell B., Tottenham N. (2014). The international society for developmental psychobiology Sackler symposium: Early adversity and the maturation of emotion circuits—A cross-species analysis. Dev. Psychobiol..

[20] Gee D.G., Gabard-Durnam L.J., Flannery J., Goff B., Humphreys K.L., Telzer E.H., Hare T.A., Bookheimer S.Y., Tottenham N. (2013). Early developmental emergence of human amygdala–prefrontal connectivity after maternal deprivation. Proc. Natl. Acad. Sci. USA.

[21] Lovallo W.R. (2013). Early life adversity reduces stress reactivity and enhances impulsive behavior: Implications for health behaviors. Int. J. Psychophysiol..

[22] Mlouki I., Bouanene I., Sioud I., Bchir A., al’Absi M., El Mhamdi S. (2021). Impulsivity mediates the impact of early life adversity on high risk behaviors among Tunisian adolescents. Prev. Med. Rep..

[23] Taylor S. (2013). Molecular genetics of obsessive–compulsive disorder: A comprehensive meta-analysis of genetic association studies. Mol. Psychiatry.

[24] Stella V.J. (2004). Prodrugs as therapeutics. Expert Opin. Ther. Pat..

[25] Boger S., Ehring T., Berberich G., Werner G.G. (2020). Impact of childhood maltreatment on obsessive-compulsive disorder symptom severity and treatment outcome. Eur. J. Psychotraumatol..

[26] Ou W., Li Z., Zheng Q., Chen W., Liu J., Liu B., Zhang Y. (2021). Association between childhood maltreatment and symptoms of obsessive-compulsive disorder: A meta-analysis. Front. Psychiatry.

[27] Destree L., Brierley M.-E.E., Albertella L., Jobson L., Fontenelle L.F. (2021). The effect of childhood trauma on the severity of obsessive-compulsive symptoms: A systematic review. J. Psychiatr. Res..

[28] Adams T.G., Kelmendi B., Brake C.A., Gruner P., Badour C.L., Pittenger C. (2018). The role of stress in the pathogenesis and maintenance of obsessive-compulsive disorder. Chronic Stress.

[29] NICHD Early Child Care Research Network (1997). The effects of infant child care on infant-mother attachment security: Results of the NICHD study of early child care. Child Dev..

[30] Bowlby J. (1944). Forty-four juvenile thieves: Their characters and home-life (II). Int. J. Psychoanal..

[31] Bowlby J. (1951). Maternal Care and Mental Health.

[32] McCall R.B., Groark C.J., Hawk B.N., Julian M.M., Merz E.C., Rosas J.M., Muhamedrahimov R.J., Palmov O.I., Nikiforova N.V. (2019). Early caregiver–child interaction and children’s development: Lessons from the St. Petersburg-USA Orphanage intervention research project. Clin. Child Fam. Psychol. Rev..

[33] Cramm H., McColl M.A., Aiken A.B., Williams A. (2019). The mental health of military-connected children: A scoping review. J. Child Fam. Stud..

[34] Anglin D.M., Cohen P.R., Chen H. (2008). Duration of early maternal separation and prediction of schizotypal symptoms from early adolescence to midlife. Schizophr. Res..

[35] Csóka S., Simor P., Szabó G., Kopp M.S., Bódizs R. (2011). Early maternal separation, nightmares, and bad dreams: Results from the Hungarostudy Epidemiological Panel. Attach. Hum. Dev..

[36] Enoch M.-A. (2011). The role of early life stress as a predictor for alcohol and drug dependence. Psychopharmacology.

[37] Toledo-Rodriguez M., Sandi C. (2007). Stress before puberty exerts a sex-and age-related impact on auditory and contextual fear conditioning in the rat. Neural Plast..

[38] Lehmann J., Pryce C.R., Bettschen D., Feldon J. (1999). The maternal separation paradigm and adult emotionality and cognition in male and female Wistar rats. Pharmacol. Biochem. Behav..

[39] Arborelius L., Eklund M.B. (2007). Both long and brief maternal separation produces persistent changes in tissue levels of brain monoamines in middle-aged female rats. Neuroscience.

[40] Sousa V.C., Vital J., Costenla A.R., Batalha V.L., Sebastião A.M., Ribeiro J.A., Lopes L. (2014). V Maternal separation impairs long term-potentiation in CA1-CA3 synapses and hippocampal-dependent memory in old rats. Neurobiol. Aging.

[41] Tractenberg S.G., Levandowski M.L., de Azeredo L.A., Orso R., Roithmann L.G., Hoffmann E.S., Brenhouse H., Grassi-Oliveira R. (2016). An overview of maternal separation effects on behavioural outcomes in mice: Evidence from a four-stage methodological systematic review. Neurosci. Biobehav. Rev..

[42] Romeo R.D., Mueller A., Sisti H.M., Ogawa S., McEwen B.S., Brake W.G. (2003). Anxiety and fear behaviors in adult male and female C57BL/6 mice are modulated by maternal separation. Horm. Behav..

[43] Miragaia A.S., de Oliveira Wertheimer G.S., Consoli A.C., Cabbia R., Longo B.M., Girardi C.E.N., Suchecki D. (2018). Maternal deprivation increases anxiety-and depressive-like behaviors in an age-dependent fashion and reduces neuropeptide Y expression in the amygdala and hippocampus of male and female young adult rats. Front. Behav. Neurosci..

[44] Millstein R.A., Holmes A. (2007). Effects of repeated maternal separation on anxiety-and depression-related phenotypes in different mouse strains. Neurosci. Biobehav. Rev..

[45] Tan S., San Ho H., Song A.Y., Low J., Je H.S. (2017). Maternal separation does not produce a significant behavioral change in mice. Exp. Neurobiol..

[46] Feng X., Wang L., Yang S., Qin D., Wang J., Li C., Lv L., Ma Y., Hu X. (2011). Maternal separation produces lasting changes in cortisol and behavior in rhesus monkeys. Proc. Natl. Acad. Sci. USA.

[47] Uysal N., Şişman A.R., Gönenç S., Acikgöz O., Kayatekin B.M., Yalaz G. (2008). Effects Of Repeated Maternal Separation On Oxidative Stress In Adolescent Male and Female Rat Brains. J. Neurol. Sci..

[48] Sullivan R.M., Opendak M. (2021). Neurobiology of infant fear and anxiety: Impacts of delayed amygdala development and attachment figure quality. Biol. Psychiatry.

[49] Hui J., Zhang Z., Liu S., Xi G., Zhang X., Teng G.-J., Chan K.C., Wu E.X., Nie B., Shan B. (2011). Hippocampal neurochemistry is involved in the behavioural effects of neonatal maternal separation and their reversal by post-weaning environmental enrichment: A magnetic resonance study. Behav. Brain Res..

[50] Grochecki P., Smaga I., Surowka P., Marszalek-Grabska M., Kalaba P., Dragacevic V., Kotlinska P., Filip M., Lubec G., Kotlinska J.H. (2022). Novel Dopamine Transporter Inhibitor, CE-123, Ameliorates Spatial Memory Deficits Induced by Maternal Separation in Adolescent Rats: Impact of Sex. Int. J. Mol. Sci..

[51] Colorado R.A., Shumake J., Conejo N.M., Gonzalez-Pardo H., Gonzalez-Lima F. (2006). Effects of maternal separation, early handling, and standard facility rearing on orienting and impulsive behavior of adolescent rats. Behav. Process..

[52] Delavari F., Sheibani V., Esmaeili-Mahani S., Nakhaee N. (2016). Maternal separation and the risk of drug abuse in later life. Addict. Health.

[53] Pellow S., File S.E. (1986). Anxiolytic and anxiogenic drug effects on exploratory activity in an elevated plus-maze: A novel test of anxiety in the rat. Pharmacol. Biochem. Behav..

[54] Handley S.L. (1991). Evaluation of marble-burying behavior as a model of anxiety. Pharmacol. Biochem. Behav..

[55] Angoa-Pérez M., Kane M.J., Briggs D.I., Francescutti D.M., Kuhn D.M. (2013). Marble burying and nestlet shredding as tests of repetitive, compulsive-like behaviors in mice. JoVE J. Vis. Exp..

[56] Thomas A., Burant A., Bui N., Graham D., Yuva-Paylor L.A., Paylor R. (2009). Marble burying reflects a repetitive and perseverative behavior more than novelty-induced anxiety. Psychopharmacology.

[57] Shin S.Y., Han S.H., Woo R.-S., Jang S.H., Min S.S. (2016). Adolescent mice show anxiety-and aggressive-like behavior and the reduction of long-term potentiation in mossy fiber-CA3 synapses after neonatal maternal separation. Neuroscience.

[58] Mahmoodkhani M., Ghasemi M., Derafshpour L., Amini M., Mehranfard N. (2020). Long-term decreases in the expression of calcineurin and GABAa receptors induced by early maternal separation are associated with increased anxiety-like behavior in adult male rats. Dev. Neurosci..

[59] Sterley T.-L., Howells F.M., Russell V.A. (2011). Effects of early life trauma are dependent on genetic predisposition: A rat study. Behav. Brain Funct..

[60] Bian Y., Ma Y., Ma Q., Yang L., Zhu Q., Li W., Meng L. (2021). Prolonged Maternal Separation Induces the Depression-like Behavior Susceptibility to Chronic Unpredictable Mild Stress Exposure in Mice. Biomed Res. Int..

[61] Lundberg S., Abelson K.S.P., Nylander I., Roman E. (2017). Few long-term consequences after prolonged maternal separation in female Wistar rats. PLoS ONE.

[62] Cruz F.C., Quadros I.M., da Planeta C.S., Miczek K.A. (2008). Maternal separation stress in male mice: Long-term increases in alcohol intake. Psychopharmacology.

[63] Jarrar B., Al-Doaiss A., Shati A., Al-Kahtani M., Jarrar Q. (2021). Behavioural alterations induced by chronic exposure to 10 nm silicon dioxide nanoparticles. IET Nanobiotechnology.

[64] Tirumalasetti J., Patel M., Shaikh U., Harini K., Shankar J. (2015). Evaluation of skeletal muscle relaxant activity of aqueous extract of Nerium oleander flowers in Albino rats. Indian J. Pharmacol..

[65] Levy S., Muench J. (2022). The epigenetic impact of adverse childhood experiences through the lens of personalized medicine. Epigenomics.

[66] Lundgaard Donovan L., Henningsen K., Flou Kristensen A., Wiborg O., Nieland J.D., Lichota J. (2021). Maternal separation followed by chronic mild stress in adulthood is associated with concerted epigenetic regulation of AP-1 complex genes. J. Pers. Med..

[67] Krause B.J., Artigas R., Sciolla A.F., Hamilton J. (2020). Epigenetic mechanisms activated by childhood adversity. Epigenomics.

[68] Thumfart K.M., Jawaid A., Bright K., Flachsmann M., Mansuy I.M. (2021). Epigenetics of childhood trauma: Long term sequelae and potential for treatment. Neurosci. Biobehav. Rev..

[69] Aisa B., Tordera R., Lasheras B., Del Río J., Ramírez M.J. (2007). Cognitive impairment associated to HPA axis hyperactivity after maternal separation in rats. Psychoneuroendocrinology.

[70] Kalinichev M., Easterling K.W., Plotsky P.M., Holtzman S.G. (2002). Long-lasting changes in stress-induced corticosterone response and anxiety-like behaviors as a consequence of neonatal maternal separation in Long–Evans rats. Pharmacol. Biochem. Behav..

[71] Lehmann J., Feldon J. (2000). Long-term biobehavioral effects of maternal separation in the rat: Consistent or confusing?. Rev. Neurosci..

[72] Winters K.C., Arria A. (2011). Adolescent brain development and drugs. Prev. Res..

[73] De Villiers M., Van den Berg H. (2012). The implementation and evaluation of a resiliency programme for children. S. Afr. J. Psychol..

[74] Scientific Council N. (2014). Excessive stress disrupts the development of brain architecture. J. Child. Serv..

[75] Liu C., Xu L., Li J., Zhou F., Yang X., Zheng X., Fu M., Li K., Sindermann C., Montag C. (2021). Serotonin and early life stress interact to shape brain architecture and anxious avoidant behavior–a TPH2 imaging genetics approach. Psychol. Med..

[76] Potter M. (1985). History of the BALB/c family. The BALB/c Mouse.

[77] Brodkin E.S. (2007). BALB/c mice: Low sociability and other phenotypes that may be relevant to autism. Behav. Brain Res..

[78] Razzoli M., Carboni L., Andreoli M., Ballottari A., Arban R. (2011). Different susceptibility to social defeat stress of BalbC and C57BL6/J mice. Behav. Brain Res..

[79] Savignac H.M., Finger B.C., Pizzo R.C., O’leary O.F., Dinan T.G., Cryan J.F. (2011). Increased sensitivity to the effects of chronic social defeat stress in an innately anxious mouse strain. Neuroscience.

[80] Kudryashov N.V., Kalinina T.S., Zhmurenko L.A., Voronina T.A. (2016). Anticompulsive activity of a new pyrazolo [C] pyridine derivative GIZh-72 under conditions of unpredictable chronic mild stress. Bull. Exp. Biol. Med..

[81] Gomes J.A.S., Oliveira M.C., Gobira P.H., Silva G.C., Marçal A.P., Gomes G.F., Ferrari C.Z., Lemos V.S., de Oliveira A.C.P., Vieira L.B. (2018). A high-refined carbohydrate diet facilitates compulsive-like behavior in mice through the nitric oxide pathway. Nitric Oxide.

[82] Ponzoni L., Braida D., Carboni L., Moretti M., Viani P., Clementi F., Zoli M., Gotti C., Sala M. (2020). Persistent cognitive and affective alterations at late withdrawal stages after long-term intermittent exposure to tobacco smoke or electronic cigarette vapour: Behavioural changes and their neurochemical correlates. Pharmacol. Res..

[83] Marchette R.C.N., Bicca M.A., da Silva Santos E.C., de Lima T.C.M. (2018). Distinctive stress sensitivity and anxiety-like behavior in female mice: Strain differences matter. Neurobiol. Stress.

[84] Biedermann S.V., Biedermann D.G., Wenzlaff F., Kurjak T., Nouri S., Auer M.K., Wiedemann K., Briken P., Haaker J., Lonsdorf T.B. (2017). An elevated plus-maze in mixed reality for studying human anxiety-related behavior. BMC Biol..

[85] Anggreini P., Ardianto C., Rahmadi M., Khotib J. (2019). Quercetin attenuates acute predator stress exposure-evoked innate fear and behavioral perturbation. J. Basic Clin. Physiol. Pharmacol..

[86] Rodgers R.J., Dalvi A. (1997). Anxiety, defence and the elevated plus-maze. Neurosci. Biobehav. Rev..

[87] Lalonde R., Strazielle C. (2010). Relations between open-field, elevated plus-maze, and emergence tests in C57BL/6J and BALB/c mice injected with GABA-and 5HT-anxiolytic agents. Fundam. Clin. Pharmacol..

[88] Brown G.R., Nemes C. (2008). The exploratory behaviour of rats in the hole-board apparatus: Is head-dipping a valid measure of neophilia?. Behav. Process..

[89] Bath K.G., Manzano-Nieves G., Goodwill H. (2016). Early life stress accelerates behavioral and neural maturation of the hippocampus in male mice. Horm. Behav..

[90] Bakhshani N.-M. (2014). Impulsivity: A predisposition toward risky behaviors. Int. J. High Risk Behav. Addict..

[91] Wilkie D.M., MacLennan A.J., Pinel J.P.J. (1979). RAT Defensive Behavior: Burying Noxious Food 1. J. Exp. Anal. Behav..

[92] Poling A., Cleary J., Monaghan M. (1981). Burying by rats in response to aversive and nonaversive stimuli. J. Exp. Anal. Behav..

[93] Taylor G.T., Lerch S., Chourbaji S. (2017). Marble burying as compulsive behaviors in male and female mice. Acta Neurobiol. Exp..

[94] Llaneza D.C., Frye C.A. (2009). Progestogens and estrogen influence impulsive burying and avoidant freezing behavior of naturally cycling and ovariectomized rats. Pharmacol. Biochem. Behav..

[95] Cservenka A., Ray L.A. (2017). Self-reported attentional and motor impulsivity are related to age at first methamphetamine use. Addict. Behav..

[96] Boger S., Ehring T., Schwarzkopf W., Werner G.G. (2020). Potential mediators of the association between childhood maltreatment and obsessive-compulsive disorder in adulthood. J. Obs. Compuls. Relat. Disord..

[97] Bowlby J. (1979). The bowlby-ainsworth attachment theory. Behav. Brain Sci..

[98] Tops M., Van Peer J.M., Korf J., Wijers A.A., Tucker D.M. (2007). Anxiety, cortisol, and attachment predict plasma oxytocin. Psychophysiology.

[99] De Weerth C., Buitelaar J.K., Beijers R. (2013). Infant cortisol and behavioral habituation to weekly maternal separations: Links with maternal prenatal cortisol and psychosocial stress. Psychoneuroendocrinology.

[100] De Weerth C., Zijlmans M.A.C., Mack S., Beijers R. (2013). Cortisol reactions to a social evaluative paradigm in 5-and 6-year-old children. Stress.

[101] Doulougeri K., Panagopoulou E., Montgomery A. (2013). The impact of maternal stress on initiation and establishment of breastfeeding. J. Neonatal Nurs..

[102] Fallon V., Groves R., Halford J.C.G., Bennett K.M., Harrold J.A. (2016). Postpartum anxiety and infant-feeding outcomes: A systematic review. J. Hum. Lact..

